# Compound heterozygous RHCE null genotype underlying D−− phenotype in a Chinese pregnant woman: molecular characterization and sequential transfusion strategy

**DOI:** 10.3389/fimmu.2026.2004196

**Published:** 2026-10-15

**Authors:** Wenjing Zhou, Li An, Lei Li, Haiyan Wan, Wangnan Sun, Qi He, Na Zhao, Mo Wang, Shouyong Hun

**Affiliations:** 1 Department of Blood Transfusion, Shandong Provincial Hospital Affiliated to Shandong First Medical University, Jinan, Shandong, China; 2 Shandong Blood Center, Jinan, Shandong, China; 3 Department of Obstetrics, Shandong Provincial Hospital Affiliated to Shandong First Medical University, Jinan, Shandong, China; 4 Department of Vascular Surgery, Shandong Provincial Hospital Affiliated to Shandong First Medical University, Jinan, Shandong, China

**Keywords:** alloimmunization, autologous transfusion, compound heterozygosity, hemolytic disease of the fetus and newborn, RHCE null allele, third-generation sequencing

## Abstract

The D−− phenotype is an extremely rare Rh blood group variant with absence of C, c, E, and e antigens but preserved D expression, predisposing to anti-Rh17 alloimmunization and hemolytic disease of fetus and newborn (HDFN). We report a 25-year-old Chinese woman (gravida 3, para 1) with D+C−c−E−e− phenotype and a history of severe neonatal jaundice and fetal demise. In her current pregnancy, admitted at 25^+5^ weeks for elevated fetal middle cerebral artery peak systolic velocity. PacBio sequencing (proband and her parents) and Oxford Nanopore whole-genome sequencing (ONT WGS; proband) were performed. PacBio sequencing preliminarily identified a compound heterozygous state at the *RHCE* locus involving two distinct *RHCE*-null haplotypes, a paternal complex structural variant and a maternal RHCE*02N.03 null allele with seven mutations. Subsequent ONT WGS resolved a novel paternal chimeric *RHD–TMEM50A–RHCE* rearrangement arising from dual recombination events. Serological and family evidence suggest that the maternal RHCE*02N.03 allele with c.486 + 1G>A results in a C−e− null phenotype in the compound heterozygous state. Four serial intrauterine transfusions used allogeneic D−− donor blood or maternal autologous washed irradiated packed red blood cells. All transfusions were well tolerated, with delivery at 37 weeks by cesarean section and favorable neonatal outcomes. This case demonstrates a sequential transfusion strategy for D−− pregnancies—prioritizing allogeneic D−− blood from rare donor networks when available, with maternal autologous blood as a safe alternative—and, through complementary PacBio and ONT WGS, adds evidence that complex *RHD-RHCE* inversion-recombination events may represent recurret mechanisms in the Chinese population while refining RHCE*02N.03 annotation from C− to C−e−.

## Highlights

- A novel paternal *RHD-RHCE* rearrangement and maternal null allele were identified in a D−− proband by integrated PacBio and ONT WGS.
- A sequential transfusion strategy using donor or maternal blood enabled successful serial intrauterine transfusions in a D−− pregnancy.

## 1 Introduction

The Rhesus (Rh) blood group system is highly polymorphic with >50 antigens encoded by the homologous *RHD* and *RHCE* genes (1, 2). These genes lie tail-to-tail, separated by TMEM50A, and share approximately 97% sequence identity (3, 4). The D, C, c, E, and e antigens are considered most clinically significant in transfusion medicine and obstetric practice (5). The D−− phenotype, characterized by complete absence of C, c, E, and e with preserved D expression, is exceptionally rare (0.005% in American Hispanics, approximately 1:100, 000 in Japan) (6, 7). In China, sporadic cases are associated with severe hemolytic disease of the fetus and newborn (HDFN) and substantial transfusion challenges (8).

The molecular mechanisms underlying the D−− phenotype include *RHD-RHCE* gene rearrangements, hybrid alleles, and splice-site mutations (2, 3, 9). Third-generation sequencing and optical genome mapping have uncovered complex inversions and recombinations spanning the *RHD, RHCE*, and interposed *TMEM50A* genes. Li et al. (3) identified a novel complex *RH* locus rearrangement involving inversion and recombination that led to the D−− phenotype in a Chinese family. Ying et al. (10) found a recurrent structural variant in six unrelated Chinese D−− individuals, suggesting that complex structural variations may be major mechanisms in the Chinese population. However, short-read and Sanger sequencing alone cannot resolve haplotype phase or large-scale structural architecture (11, 12).

Individuals with D−− phenotype are at risk of anti-Rh17 alloimmunization upon exposure to RhCE antigens through pregnancy or transfusion (13). These antibodies react with all common Rh phenotypes except Rhnull and D−− cells, complicating transfusion and pregnancy management (14, 15). Anti-Rh17-mediated HDFN can cause severe fetal anemia, hydrops fetalis, or fetal death (13, 16); more than 90% of affected pregnancies require intrauterine transfusion (IUT), often starting in the second trimester (13). Finding compatible blood for IUT in D−− pregnancies is difficult due to the scarcity of D−− donors. Once identified, individuals with the D−− phenotype should be enrolled in a rare blood donor registry available for donation (17). In emergency settings without compatible donors, maternal autologous blood serves as a viable alternative (16).

In this study, we report molecular characterization of a Chinese women with the D−− phenotype using integrated PacBio and Oxford Nanopore Technologies whole-genome sequencing (ONT WGS) with family studies. We further describe the successful management of severe HDFN using a sequential allogeneic-autologous transfusion strategy and propose a management algorithm that emphasizes the central role of the transfusion department.

## 2 Methods

### 2.1 Study design

This was a single-case molecular and clinical investigation of a pregnant woman with the D−− phenotype and her family members. The study was approved by the Institutional Ethics Committee of Shandong Provincial Hospital Affiliated to Shandong First Medical University (approval number: 2026-878). Written informed consent was obtained from all participants. Separate written informed consent was obtained for genetic testing, blood sampling, and intrauterine transfusion procedures.

### 2.2 Study subject

A 25-year-old Chinese woman (gravida 3, para 1) was referred to our transfusion department for management of her third pregnancy. She was admitted at 25^+5^ weeks of gestation due to elevated fetal middle cerebral artery peak systolic velocity. Peripheral whole blood samples were obtained from the proband, her parents, and her husband. In addition, cord blood was collected prior to the intrauterine transfusion procedure. Diagnostic cord blood sampling was performed on day 3 of hospitalization; the first IUT was performed at 26^+6^ weeks after maternal autologous blood preparation.

### 2.3 Serological testing

Red blood cells from the proband, parents, husband, and fetal cord (2%-5%) were tested. ABO typing used both the conventional tube method and microcolumn gel cards with monoclonal anti-A and anti-B reagents (Shanghai Hemo-Pharmaceutical & Biological Co., Ltd., Shanghai, China; lot no. 20250711). Rh phenotyping used a commercial monoclonal antibody card containing anti-D, anti-C, anti-c, anti-E, and anti-e (Changchun Bioxun Biotechnology Co., Ltd., Changchun, China; lot no. 20251103). Antibody screening and identification were performed using panel cells (ANTIGRAM antigen profile, Sanquin, Amsterdam, the Netherlands; lot no. 3SS808) and reverse typing cells (Ortho-Clinical Diagnostics, Raritan, NJ, USA; lot no. A0322), analyzed by gel cards.

### 2.4 PacBio long-read sequencing and family-based validation

Targeted long-read sequencing of full-length *RHD*, *RHCE*, and *RHAG* was performed on the PacBio Revio platform (Pacific Biosciences, USA) by Xi’An Haorui Genomics Technologies Ltd. Genomic DNA from peripheral blood was extracted (MGIEasy kit, MGI, China). *RH* genes were amplified as overlapping amplicons (7 for *RHD*, 8 for *RHCE*) using long-range PCR for haplotype assembly. Libraries were constructed following the manufacturer’s instructions (Haorui Genomics, China) and sequenced on the Revio platform. Primary data were processed with SMRTLink v26.1 software (Pacific Biosciences, USA) to generate HiFi reads, which are high-accuracy sequences with a quality score over Q30 (99.9% accuracy). Filtered CCS reads were aligned to GRCh38 using pbmm2 (v1.16.0). Reads covering *RHD, RHCE*, and *RHAG* regions of interest were further aligned against International Society of Blood Transfusion (ISBT) reference sequences of *RHD* (NG_007494.1), *RHCE* (NG_009208.3), and *RHAG* (NG_011704.1). DeepVariant (v1.6.1) was employed for SNV, indel, and variant calling analysis. Integrative Genomics Viewer and SnapGene (GSL Biotech LLC, USA) were used for visualization and manual validation. Haplotype phasing and co-segregation analysis used family-based segregation analysis combined with PacBio CCS read clustering, assigning reads to parental haplotypes based on private variants (≥10 reads per heterozygous site). Structural junctions and mutations were supported by PacBio data where amplicon coverage was available, and by family co-segregation analysis (3, 10).

### 2.5 Whole-genome sequencing for proband

Whole-genome sequencing (WGS) was performed on the proband using the Oxford Nanopore PromethION platform (ONT, UK) to fully resolve the structure of the paternal allele and to provide genome-wide validation of structural variations. The SQK-LSK114 protocol for library preparation and sequencing was performed according to the manufacturer’s instructions. Briefly, DNA template damage and ends were repaired in a combined step, followed by AMPureXP bead purification (Beckman Coulter, CA) and ligation of platform-specific adapter sequences. The flow cell premix was prepared with EXP-FLP001 PRO.6 sequencing chip preparation kit, and the final library was loaded onto PromethION R10.4 flow cell (FLO-PRO114M) for nanopore sequencing following the manufacturer’s instructions. Base calling of the raw reads was performed using ONT basecaller Guppy (v1.4.0). The reads were aligned to the human reference genome (CHM13v2.0) using minimap2 (v2.14-r883) with default parameters. The sequencing run generated 132.13 Gb of data comprising 23.10 million reads, with an average read length of >30 kb and an average whole-genome coverage depth of approximately 42×. Structural variations, including inversions, were detected using Sniffles (v2.0). Copy number variations were analyzed using CNVkit. Coverage was assessed using mosdepth, and the breakpoints and junctions of structural rearrangements were identified manually using Integrative Genomics Viewer software.

### 2.6 Obstetric and transfusion clinical procedures

#### 2.6.1 Fetal monitoring

Fetal middle cerebral artery peak systolic velocity (MCA-PSV) measurements were performed by ultrasound. MCA-PSV values were converted to multiples of the median (MoM) according to gestational age. MCA-PSV ≥1.5 MoM was considered indicative of moderate-to-severe fetal anemia and triggered fetal blood sampling (18, 19).

#### 2.6.2 Autologous blood collection and processing

Each autologous collection (200 mL whole blood, CPDA-1) was processed into washed, irradiated pRBCs (Hct ~75-80%) per standard protocols. Units were tested for infectious markers, cross-matched with maternal serum, leukoreduced, and stored at 4 °C. After each collection, the patient received iron, vitamin B12, folic acid, and erythropoietin to support erythropoiesis. Blood was collected at a rate of 50–80 mL/min under continuous monitoring of maternal vital signs and fetal heart rate. Collection was deferred if pre-collection hemoglobin was <10 g/dL or if the patient reported symptoms of acute illness. Collections were performed at least 7 days apart to allow adequate erythropoiesis.

#### 2.6.3 Intrauterine transfusion procedure

Intrauterine transfusions were performed by obstetricians via ultrasound-guided cordocentesis using a 20-gauge or 22-gauge spinal needle (20). The interval between diagnostic cordocentesis and the first IUT was approximately 1.5 weeks, reflecting the time required for autologous blood collection, processing, and quality control. Fetal hemoglobin was re-measured immediately before the first IUT; the value was 8.7 g/dL, compared with 8.9 g/dL at diagnostic cordocentesis. The diagnostic procedure carried a small inherent risk of exacerbating fetal anemia, but the repeat measurement indicated that fetal anemia had not worsened substantially during the interval. Fetal heart rate was continuously monitored and recorded every 5 minutes during the procedure. The transfusion volume was calculated using the formula: [estimated fetal weight (kg) × desired Hb increment (g/dL) × 0.08]/pRBC hematocrit (%). Post-transfusion fetal monitoring included ultrasound assessment within 24 hours and serial MCA-PSV measurements weekly thereafter (21).

### 2.7 Data analysis

Sequencing data were analyzed as described above. Clinical data, including MCA-PSV measurements, fetal hemoglobin levels, transfusion volumes, maternal hemoglobin levels, and neonatal outcomes, were recorded prospectively. All authors had full access to the primary clinical and sequencing data and participated in the interpretation of the results. Raw data are deposited in NCBI SRA (PRJNA1521709) and will be publicly available upon publication.

## 3 Results

### 3.1 Serological identification and clinical presentation

Initial immunohematological workup of the proband revealed blood group B and Rh phenotype D+C−c−E−e− (D−−). Antibody screening was strongly positive, with reactivity against all three panel cells. Antibody specificity was identified as anti-Rh17, with reactivity against red blood cells expressing C, c, E, or e antigens, consistent with the proband’s D-- phenotype. The IgG anti-Rh17 titer was 512 (microcolumn gel method), measured at the time of admission (25^+5^ weeks). Serial monitoring was not performed. A standardized reference serum was used according to the microcolumn gel protocol. This titer indicated a high-risk alloimmunization state, although the correlation between anti-Rh17 titer and fetal anemia severity is not as well established as for anti-D. The direct antiglobulin test was negative. The proband’s first pregnancy at age 20 resulted in a term delivery at 38 weeks of gestation, with the neonate developing severe jaundice requiring exchange transfusion (the infant is currently healthy); her second pregnancy at age 22 ended in intrauterine fetal demise at 13 weeks of gestation with subsequent induced labor. In the current pregnancy, she was admitted at 25^+5^ weeks for elevated MCA-PSV. Primary alloimmunization timing was unknown due to lack of prior records.

The parents had identical Rh phenotypes: D+C+c−E−e+ (father: AB; mother: B). The husband was B, D+C+c+E+e+ (**Table 1**). This phenotype in each parent reflects the presence of a functional *RHCE* allele encoding C and e, together with a *RHCE* null allele that produces no RhCE antigens. This parental phenotype—each parent lacking c and E but retaining C and e—combined with the proband’s complete absence of all RhCE antigens, supports autosomal recessive inheritance of two distinct *RHCE* null alleles. Following the identification of the D−− phenotype, the patient was registered in the national rare blood bank database. Through this network, a D−− volunteer donor was identified in Shanghai, China, who consented to blood donation for the patient’s intrauterine transfusion.

**Table 1:** Molecular findings in the proband and family members.

			Predicted by targeted long-read sequencing		
Member	ABO phenotype	Rh phenotype	Sequencing platform	*RHCE* allele 1 (paternal origin)	*RHCE* allele 2 (maternal origin)
Proband	B	D+C−c−E−e−	PacBio TGS	Complex structural variant (incompletely resolved)	RHCE*02N.03 (seven mutations)
Father	AB	D+C+c−E−e+	PacBio TGS	Complex structural variant (incompletely resolved)	Normal *RHCE* allele
Mother	B	D+C+c−E−e+	PacBio TGS	Normal *RHCE* allele	RHCE*02N.03 (seven mutations)
Husband	B	D+C+c+E+e+	Serology only	Normal *RHCE* allele (inferred from serology, not sequenced)	Normal *RHCE* allele (inferred from serology, not sequenced)

### 3.2 Preliminary detection of complex *RHCE* structural rearrangement by PacBio targeted sequencing

PacBio targeted long-read sequencing of the proband and her parents generated high-quality CCS reads with an average coverage depth of >50× across the RH gene locus. Haplotype assembly preliminarily detected a compound heterozygous status at the *RHCE* locus (two distinct *RHCE*-null haplotypes), with the paternal allele showing evidence of complex structural variation that could not be fully resolved at this stage and the maternal allele identified as RHCE*02N.03. **Table 1** summarizes the molecular findings in the proband and her family members, including the phased haplotypes, identified variants, and the inferred parental origin of each allele.

The paternal allele exhibited complex rearrangements involving *RHD-RHCE* recombination (**Figure 1**). Sequences downstream of *RHCE* exon 3 and upstream of *RHCE* exon 7 aligned to the *RHD* gene, suggesting recombination. Three copies of *RHCE* exon 8 were detected, with the third copy’s upstream sequence aligning to *RHD*, indicating copy number variation. Read-depth imbalance was observed in the *RHD* exon 4–5. Collectively, these findings indicate complex structural rearrangements in the *RHD* and *RHCE* regions of the paternal allele. Nevertheless, the targeted amplicon design, which only covers the RH locus and its immediate flanking regions, failed to fully resolve the precise exon composition, fragment order, inversion orientation, and rearrangement breakpoints.

**Figure 1:**
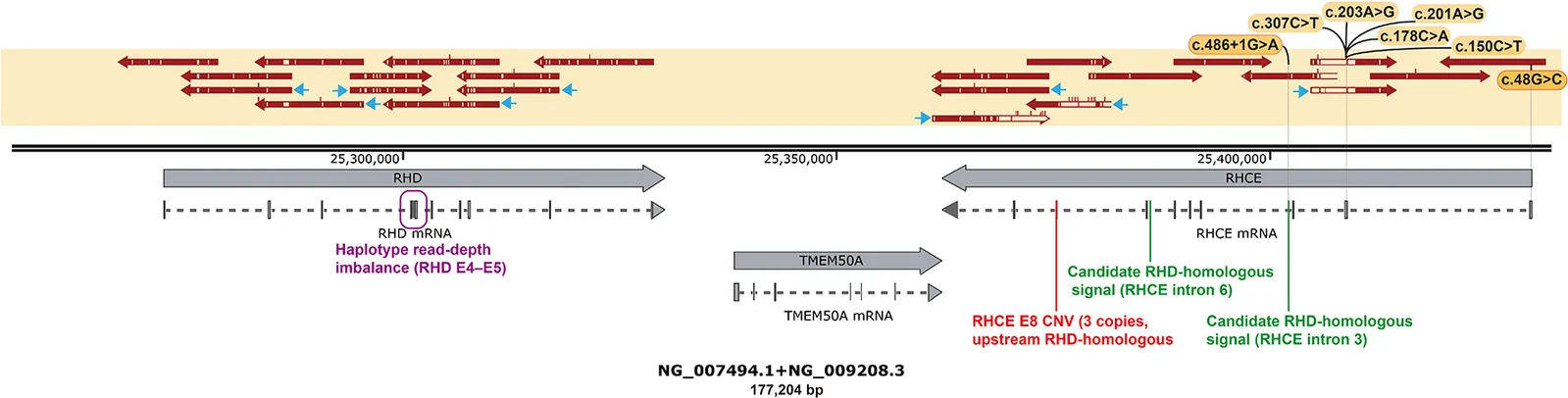
PacBio targeted sequencing identified complex structural variation of the paternal haplotype and seven single-nucleotide variants in the maternal RHCE*02N.03 allele. The combined amplicon coverage profile of both paternal and maternal haplotypes within the RH gene cluster (NG_007494.1 + NG_009208.3) is shown. Light blue small arrowheads overlay aligned reads originating from the paternal haplotype; aligned reads without light blue arrowheads correspond to the maternal haplotype. Analysis of phased long reads from the paternal allele revealed multiple lines of evidence supporting complex *RHD–RHCE* structural rearrangement: *RHD*-homologous sequences were detected downstream of *RHCE* exon 3 and upstream of *RHCE* exon 7; three copies of *RHCE* exon 8 were present, with the upstream sequence of the third copy aligning to *RHD*; and haplotype read-depth imbalance was observed across *RHD* exons 4–5. The targeted amplicon panel could not resolve the precise exon arrangement, fragment order, inversion orientation, or exact breakpoints; the definitive structural architecture of this allele was subsequently resolved by WGS.

The maternal allele was RHCE*02N.03 with seven mutations: c.48G>C; c.150C>T; c.178C>A; c.201A>G; c.203A>G; c.307C>T; c.486 + 1G>A (**Figure 1**). The c.486 + 1G>A splice variant is notable: ISBT classifies RHCE*02N.03 as C−, but serology showed the proband lacked both C and e (C−e−). Maternal inheritance of this allele—with the mother phenotypically C+e+ (functional *RHCE* allele on her other chromosome)—combined with the proband’s complete absence of C and e, suggest that c.486 + 1G>A may produce a C−e− null phenotype in the compound heterozygous state. The additional mutations further support its null nature, consistent with reported *RHCE* null alleles (22).

### 3.3 Full characterization of the paternal rearrangement haplotype by ONT WGS

To fully resolve the architecture of the complex paternal RH variant, ONT WGS combined with haplotype-resolved Integrative Genomics Viewer (IGV) visualization was performed (**Figure 2**). **Figure 2A** displays the full sequencing coverage and split-haplotype alignment spanning the entire chr1 *RHD-TMEM50A-RHCE* gene cluster. The ONT long-read data resolved the previously ambiguous regions, revealing two independent recombination signatures: one intragenic rearrangement footprint within the *TMEM50A* locus (marked by the red arrow), and a second intergenic recombination zone spanning the *RHD–RHCE* interval (marked by the blue arrow), corresponding to the canonical *RHCE-D(2–7)-CE* gene conversion event. To pinpoint the exact breakpoints of the *TMEM50A* inversion, we generated a magnified view of this genomic interval (**Figure 2B**). Two distinct sharp copy number shift breakpoints flanking the *TMEM50A* gene confirmed the existence of an inverted *TMEM50A* fragment covering exons 7 to 3. Integrating long-read haplotype partitioning and structural variant calling data, we defined the full linear structure of the abnormal paternal chimeric haplotype for the first time: *RHD(1-9)–RHCE(10)–TMEM50A(7-3)–TMEM50A–RHCE(10-8)–RHD(7-2)–RHCE(1).* We infer that this multi-gene complex rearrangement may have arisen from two separate homologous recombination events: intramolecular inversion within the *TMEM50A* gene locus, and intergenic homologous gene conversion between *RHD* and *RHCE* involving exons 2 to 7. This mechanism was inferred from the genomic structure rather than directly demonstrated.

**Figure 2:**
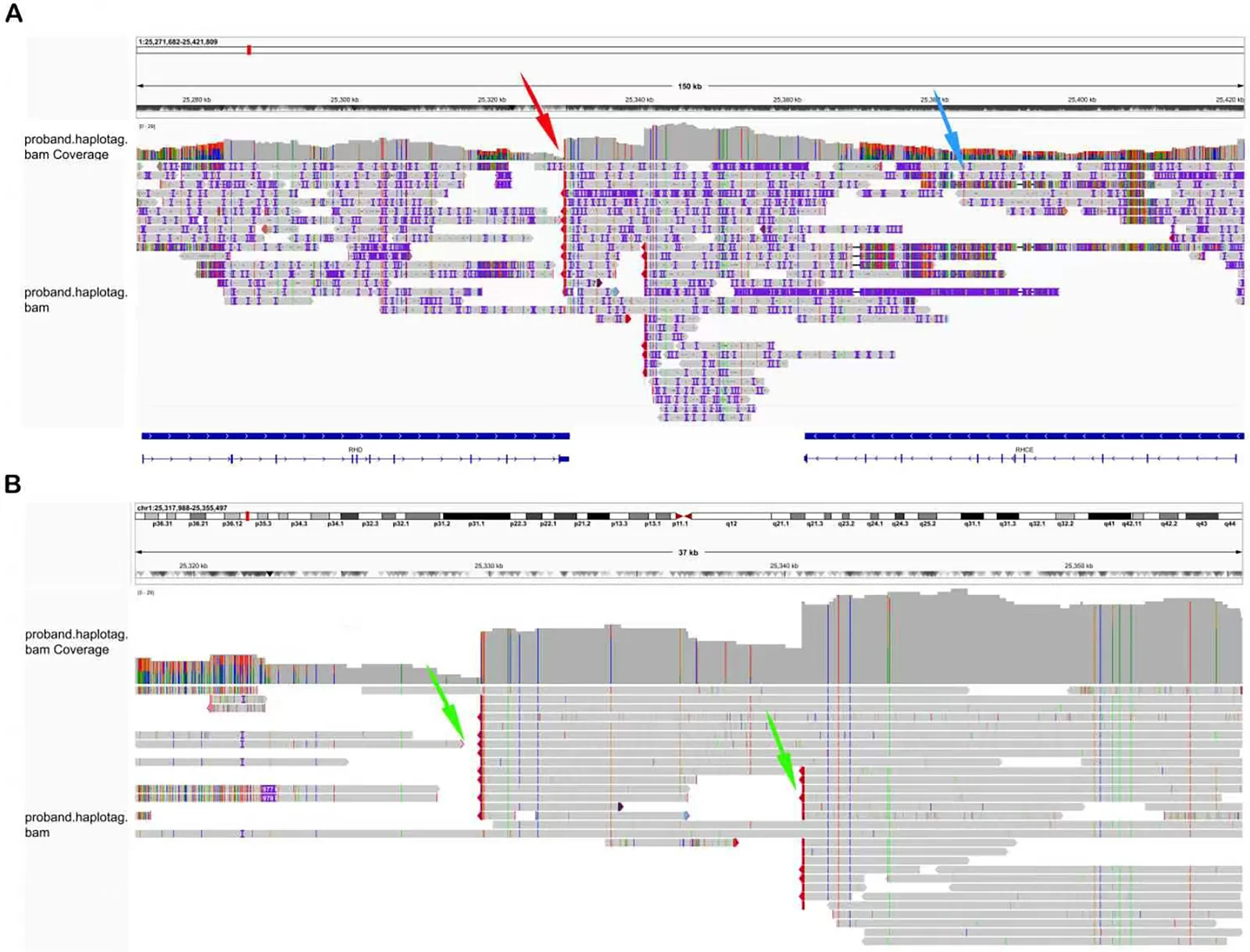
ONT WGS and haplotype-resolved IGV visualization characterize the complex multi-gene chimeric rearrangement involving *RHD, RHCE*, and *TMEM50A*. **(A)** Full-length sequencing coverage and split-haplotype alignment across the chr1 *RHD-TMEM50A-RHCE* gene cluster. The red arrow denotes the intragenic recombination signature within the *TMEM50A* locus, while the blue arrow labels the intergenic *RHCE-D(2-7)-CE* homologous recombination footprint between *RHD* and *RHCE*. **(B)** Magnified IGV view of the *TMEM50A* interval showing two distinct flanking copy number variant breakpoints, which confirm the inverted *TMEM50A* fragment covering exons 7-3.

Based on ONT WGS assembly, the full linear architecture of this paternal variant was further summarized in a schematic diagram (**Figure 3B**; **Figure 3A** illustrates the wild-type reference *RH-TMEM50A* locus). This complex rearrangement harbors two discrete clusters of recombination breakpoints, which are validated by the IGV raw data in **Figure 2** and outlined within the red dashed box in **Figure 3B**: (1) intron 2 and intron 7 breakpoints that mediate intergenic *RHCE-D(2–7)-CE* exon exchange between *RHD* and *RHCE*; (2) dual flanking copy number breakpoints at the *TMEM50A* locus that drive formation of the inverted *TMEM50A* (exons 7–3) segment.

**Figure 3:**
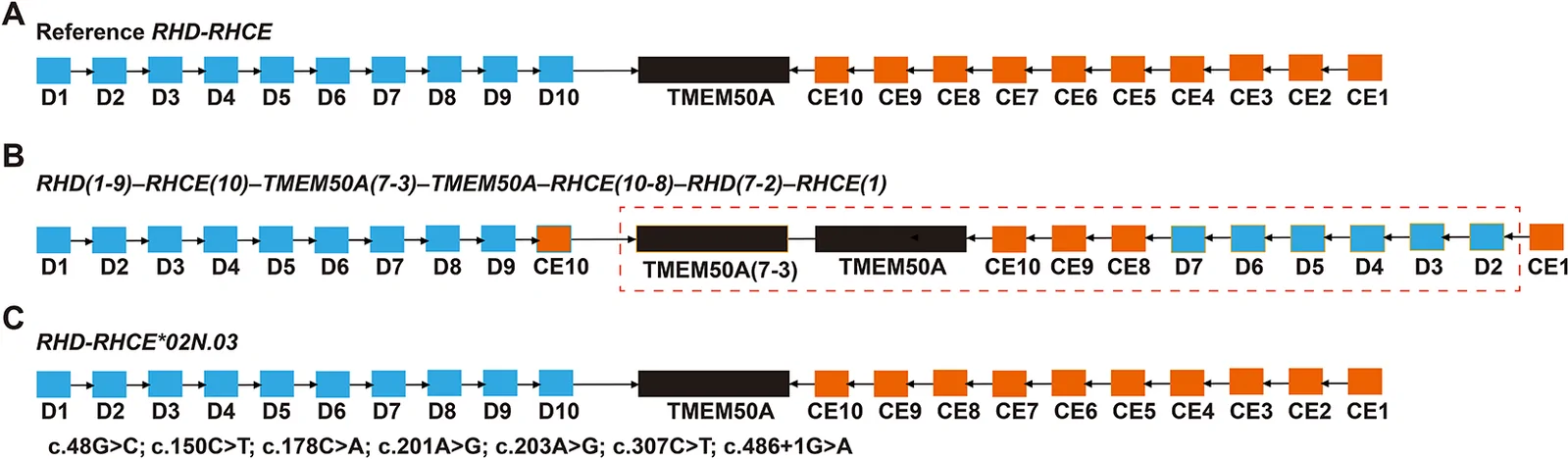
Schematic diagrams of wild-type and variant haplotypes at the *RHD-TMEM50A-RHCE* locus in this compound heterozygous D−− proband. **(A)** Wild-type reference haplotype. Blue boxes represent *RHD* exons (D1–D10); orange boxes represent *RHCE* exons (CE1–CE10); black rectangles represent *TMEM50A*. **(B)** Novel paternal chimeric rearrangement haplotype: *RHD(1-9)–RHCE(10)–TMEM50A(7-3)–TMEM50A–RHCE(10-8)–RHD(7-2)–RHCE(1).* Red dashed boxes indicate fusion regions. Red arrows denote inverted (reverse-oriented) segments.**(C)** Maternal RHCE*02N.03 null allele with seven mutations (c.48G>C; c.150C>T; c.178C>A; c.201A>G; c.203A>G; c.307C>T; c.486 + 1G>A) identified by PacBio targeted sequencing. Red arrows indicate the positions of the seven mutations. Black arrows indicate transcriptional orientation.

Notably, the inverted non-contiguous *RHD*(2–7) fragment embedded within this chimeric allele represents an unreported recombination pattern. Unlike all previously published D−− associated structural variants characterized by continuous exon exchanges (including RHD(3–8), RHD(3–9), and the RHCE*Ce(1–2)-D(3–10)-Ce(10–8)-Ce(3–10) allele described by Li et al. (3)), the inverted discontinuous insertion of *RHD* exons 7–2 identified here, to our knowledge, has not been previously reported in any known RH haplotype to date. This unique structural feature substantially expands the spectrum of complex RH gene inversion-recombination variants responsible for the D−− phenotype in human populations.

### 3.4 Family studies

Family studies confirmed the inheritance pattern: the proband inherited the paternal rearrangement haplotype from her father and the RHCE*02N.03 null allele from her mother (**Figure 4**). Serological testing confirmed that both parents had identical Rh phenotypes (D+C+c−E−e+), consistent with carrier status for a single *RHCE* null allele. Combined with the proband’s absence of all RhCE antigens, this supports autosomal recessive inheritance of two distinct null alleles. The structural junctions and mutations were validated by phased PacBio long-read sequencing data in the proband and her parents, confirming inheritance of the two recombinant haplotypes. The family pedigree is shown in **Figure 4**.

**Figure 4:**
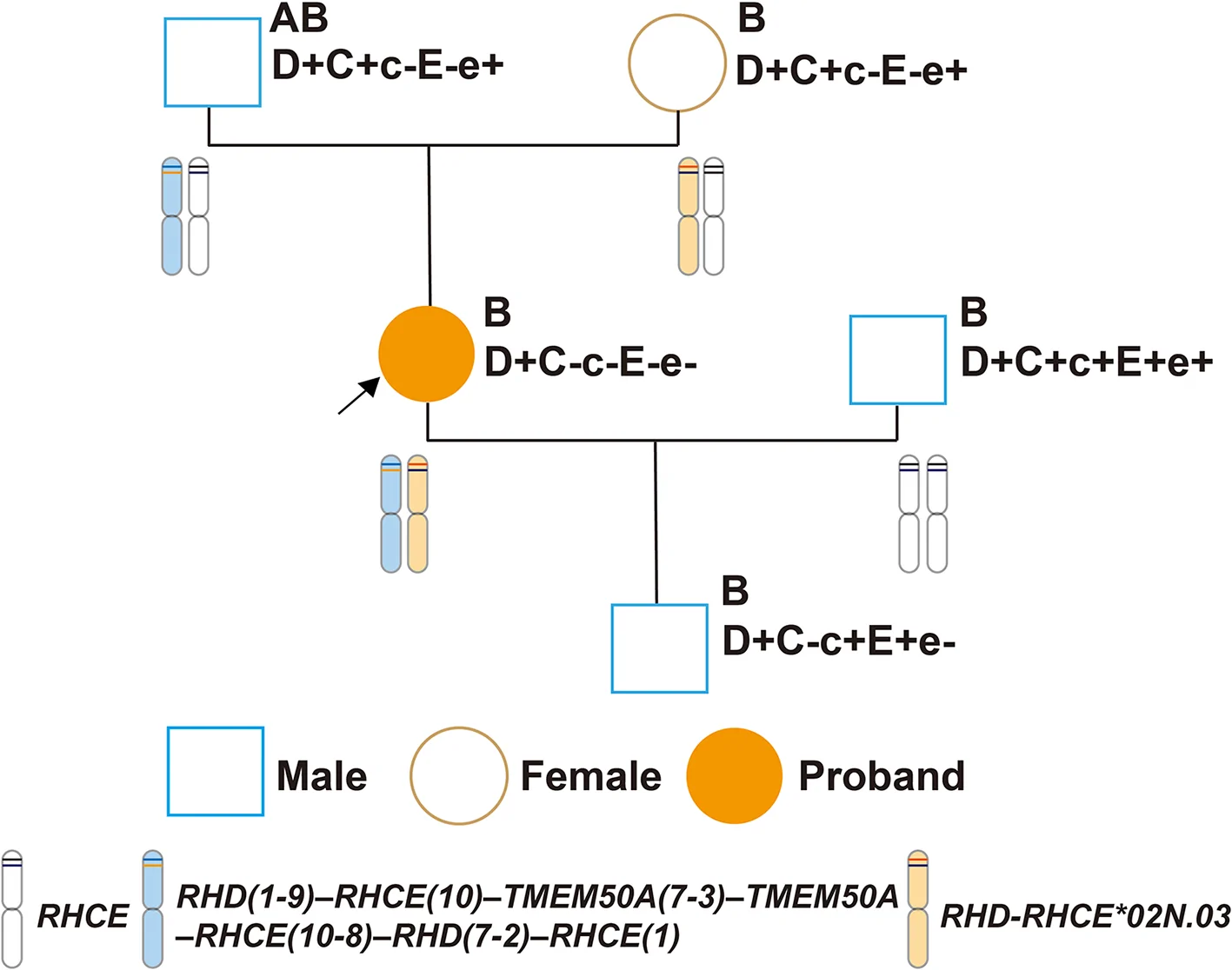
Pedigree tree with RH haplotype inheritance. Family pedigree showing the proband, her parents, husband, and child. The parents exhibit D+C+c−E−e+ phenotypes, consistent with carrier status for a single *RHCE* null allele. The proband (fully filled circle) exhibits the D+C−c−E−e− (D−−) phenotype and inherited the paternal complex structural rearrangement (*RHD(1-9)–RHCE(10)–TMEM50A(7-3)–TMEM50A–RHCE(10-8)–RHD(7-2)–RHCE(1)*) and the maternal RHCE*02N.03 null allele. The husband has a normal Rh phenotype (D+C+c+E+e+), and the child inherited paternal c and E antigens, with cord blood phenotype D+C−c+E+e−. Standard genetic symbols are used: squares = males, circles = females; filled circle = affected proband; half-filled symbols = carriers; empty symbols = unaffected. Arrows indicate inheritance of the two distinct recombinant haplotypes from each parent to the proband. Serological phenotypes of the husband and child were determined by serological testing only; molecular sequencing was not performed for these individuals.

### 3.5 Antenatal management and outcomes

Key fetal phenotype and antigen expression: Diagnostic cord blood sampling at cordocentesis confirmed the fetal group B Rh phenotype as D+C−c+E+e−, reflecting inheritance of paternal c and E antigens (father: D+C+c+E+e+) on a maternal C−E− background. Fetal expression of c and E (while C and e are absent) provides the serological basis for anti-Rh17-mediated HDFN: fetal RhCE epitopes are recognized by maternal anti-Rh17 (titer 512), causing progressive hemolytic anemia. This phenotypic finding, together with the high anti-Rh17 titer, explains the recurrent severe fetal anemia observed throughout gestation.

Fetal surveillance: Serial ultrasound assessments, including fetal MCA-PSV measurements, were performed weekly from 25^+5^ weeks of gestation. MCA-PSV values exceeding 1.5 MoM for gestational age prompted fetal blood sampling (**Figure 5**). The delay between diagnosis and the first IUT was due to the time required to prepare maternal autologous blood, as no compatible D−− donor blood was immediately available. If donor blood had been available, earlier IUT might have been considered.

**Figure 5:**
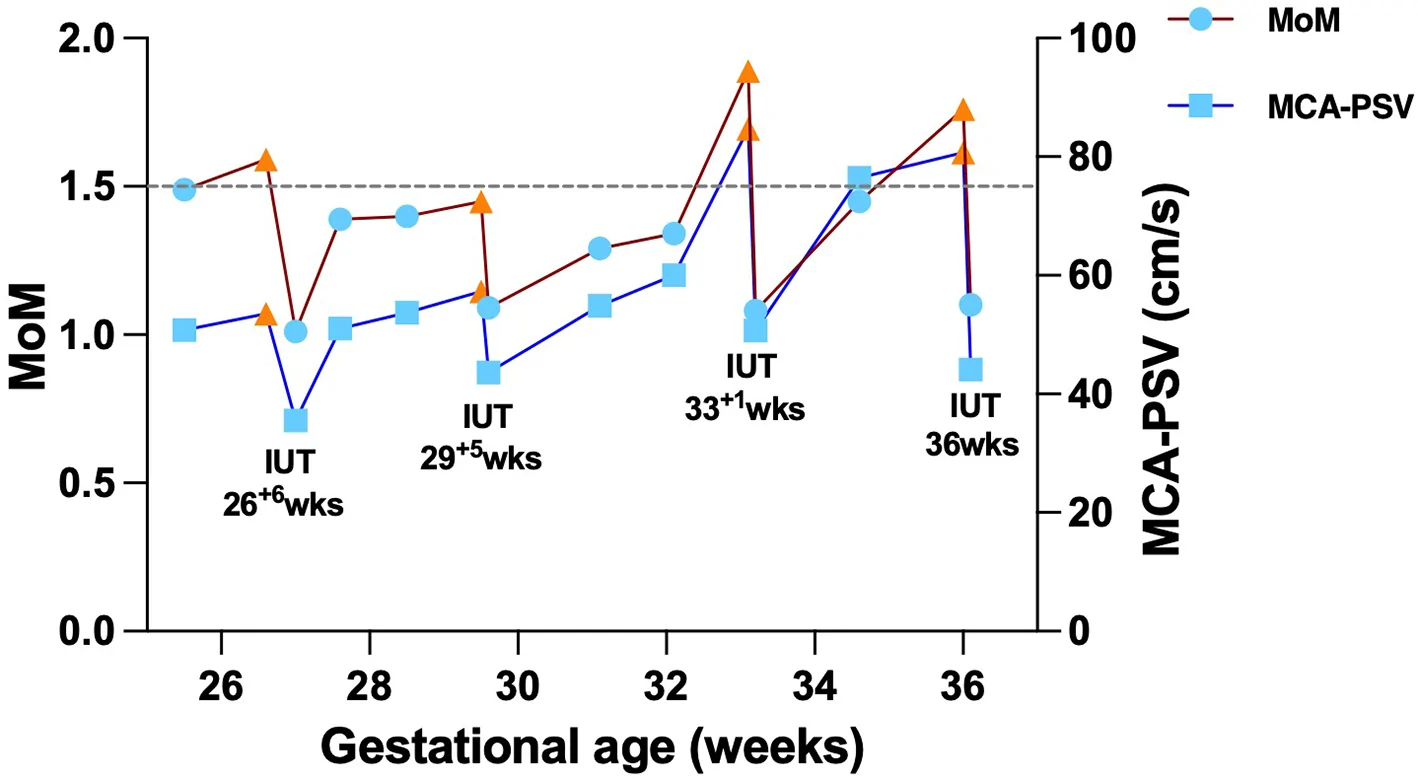
Longitudinal trends of fetal middle cerebral artery peak systolic velocity (MCA-PSV, right Y-axis) and corresponding multiples of the median (MoM, left Y-axis) across gestation. Serial MCA-PSV measurements were obtained from 25^+5^ weeks to 36 weeks of gestation. The horizontal grey dashed line denotes the 1.5 MoM threshold, indicating high risk of moderate-to-severe fetal anemia. Orange triangular markers indicate the timing of four serial intrauterine transfusions (IUTs), administered at 26^+6^, 29^+5^, 33^+1^, and 36 weeks of gestation. After each transfusion, MCA-PSV and MoM values decreased sharply, followed by gradual increases as fetal anemia recurred.

Intrauterine transfusions: Upon admission at 25^+5^ weeks of gestation, fetal umbilical cord blood sampling was performed on day 3 of hospitalization, confirming severe fetal anemia (hemoglobin 8.9 g/dL, hematocrit 27.2%). As no compatible D−− blood was immediately available, an autologous blood collection protocol was initiated. Immediately before the first IUT at 26 + 6 weeks, repeat fetal blood testing showed hemoglobin 8.7 g/dL and hematocrit 26.8%, indicating that fetal anemia had not worsened substantially during the 1.5-week interval. Four serial IUTs were performed at 26^+6^, 29^+5^, 33^+1^, and 36 weeks (**Supplementary Table 1**). The first IUT used maternal autologous blood (pre-collection hemoglobin 10.7 g/dL), the second used D−− volunteer donor blood from Shanghai, and the third and fourth used maternal autologous blood (pre-collection hemoglobin 11.7 g/dL for both). After each transfusion, fetal Hb increased from 8.9, 7.5, 7.7, and 9.4 g/dL to 13.4, 13.8, 14.5, and 14.5 g/dL, respectively. All procedures were well tolerated with no procedure-related complications. Cord blood testing confirmed the infant’s blood group as B and Rh phenotype as D+C−c+E+e−, reflecting inheritance of paternal c and E antigens (father: D+C+c+E+e+) and confirming the fetal RhCE antigen expression that triggered maternal anti-Rh17 alloimmunization. The patient delivered at 37 weeks of gestation by cesarean section. The neonate had a birth weight of 2820 g and Apgar scores of 9 at 1 minute (1 point deducted for skin color), 10 at 5 minutes, and 10 at 10 minutes. The amniotic fluid, placenta, and umbilical cord showed no abnormalities. Physical examination revealed a term-appearing infant with fair responsiveness and generalized jaundice. Neonatal laboratory findings on the day of birth included hemoglobin 13.7 g/dL, total bilirubin 105.88 μmol/L (6.19 mg/dL), direct bilirubin 14.76 μmol/L, and indirect bilirubin 91.12 μmol/L. The direct antiglobulin test was positive, and both the antibody release test and free antibody test were positive; anti-Rh17 antibody was detected in the neonatal blood. The neonate received intravenous immunoglobulin for 3 days, phototherapy for 9 days, and alkaline fluid supplementation to promote bilirubin excretion. Exchange transfusion was not required.

Maternal monitoring: Maternal hemoglobin and hematocrit were monitored before and after each autologous blood collection. Post-collection vital signs (blood pressure, heart rate) were recorded, and patients were assessed for symptoms of hypovolemia including dizziness, fatigue, and syncope. No significant adverse events occurred: all collections were completed without hypotension (systolic blood pressure <90 mmHg), tachycardia (>120 bpm), or vasovagal reactions. Pre-collection values were 10.7 g/dL (Hct 31.8%) for the first collection, 11.7 g/dL (Hct 34.0%) for the third, and 11.7 g/dL (Hct 34.5%) for the fourth.

## 4 Discussion

In this study, we report comprehensive molecular characterization of a Chinese pregnant woman with the D−− phenotype using integrated PacBio and ONT WGS, as well as the successful management of severe HDFN with a sequential allogeneic-autologous transfusion strategy. Our findings provide insights into the molecular mechanisms of the D−− phenotype and practical management strategies for rare pregnancies.

Our integrated approach revealed compound heterozygosity at the *RHCE* locus: a paternal complex rearrangement haplotype and a maternal RHCE*02N.03 null allele. PacBio detected complex structural variation on the paternal allele that could not be resolved by amplicon design alone; ONT WGS subsequently established its definitive structure. The paternal allele *RHD(1-9)–RHCE(10)–TMEM50A(7-3)–TMEM50A–RHCE(10-8)–RHD(7-2)–RHCE(1)* arises from dual independent recombination events: intramolecular inversion within the *TMEM50A* locus and intergenic inversion-conversion between *RHD* and *RHCE*. This haplotype shares similarities with previously described D−− hybrid alleles (9, 10). However, it contains a non-contiguous *RHD(7-2)* insertion—a recombination pattern not previously reported—thereby expanding the known spectrum of D−−-associated *RH* rearrangements. Such structural variants likely result in the complete absence of functional RhCE protein, highlighting the limitation of Sanger sequencing for detecting such complex variations, as observed in other D−− cases (3, 4, 10). Identification of this novel structural variant in a Chinese patient is consistent with previous reports suggesting that inversion-recombination may present a recurrent mechanism in the Chinese D−− population. However, based on a single case, conclusions about population prevalence cannot be drawn. The parental and proband phenotypes confirm autosomal recessive inheritance of biallelic defective *RHCE* alleles. No identical rearrangement was retrieved from the ISBT RH allele database, ClinVar and PubMed, and ongoing monitoring is needed as these databases update.

The maternal c.486 + 1G>A is classified by ISBT as C−, but its e antigen status is not specified (23). Through comprehensive serological testing and family analysis, we provide evidence that the RHCE*02N.03 allele with c.486 + 1G>A may result in a C−e− null phenotype, thereby refining the current ISBT annotation. However, RNA-level confirmation was not performed, and the absence of C and e cannot be unequivocally attributed to this allele alone. Unlike anti-D, the correlation between anti-Rh17 titer and fetal anemia severity is not well established; therefore, the titer should not be used alone to predict HDFN severity (24). Serology confirmed the proband lacked both C and e, with no weak e expression (excluded by multiple anti-e monoclonal antibodies, clones MS-16, MS-21, MS-63). The proband inherited this allele from her C+e+ mother (a functional *RHCE* on the other allele), and the paternal allele lacked both C and e; thus, the proband’s C−e− phenotype results from two null alleles. The c.486 + 1G>A mutation disrupts the invariant GT donor splice site of intron 4 (25), eliminating the GU donor sequence required for spliceosome recognition (26). This predicts exon 4 skipping, cryptic splice site activation, or intron retention—all leading to frameshift and non-functional RhCE protein. When RHCE*02N.03 is heterozygous with a normal allele (as in the proband’s mother), the e antigen from the normal allele masks the null phenotype; complete loss of e is revealed only in compound heterozygous or homozygous states. These findings have implications for genotyping and alloimmunization risk prediction. Although confirmation in additional cases is needed before ISBT revision, our case provides data supporting refining RHCE*02N.03 to C−e− null phenotype (27).

This study highlights the complementary value of two distinct long-read sequencing strategies. PacBio targeted sequencing provided high-depth phased haplotype, enabling family-based haplotype phasing and preliminary detection of compound heterozygosity at the *RHCE* locus (3). It resolved the maternal RHCE*02N.03 null allele and provided critical evidence of paternal structural variation (*RHCE-D(2-7)-RHCE* recombination, intron 7 variation, and RHCE exon 8 copy number variation). However, PacBio amplicons did not span *TMEM50A*, where inversion breakpoints were later identified. This limitation was overcome by ONT WGS, whose long reads (10–100 kb) provided continuous coverage across the entire *RHD-TMEM50A-RHCE* interval (3, 11). The combination of these two platforms addresses their respective limitations. PacBio targeted sequencing offers higher accuracy for point mutations and small indels but may miss large-scale structural variations if target regions are not optimally designed (4). ONT WGS provides broader coverage and better detection of large-scale rearrangements but may have lower single-base accuracy and higher sequencing cost for high-depth coverage (11). Their integration—PacBio for family-based haplotype phasing and mutation detection, ONT for genome-wide structural resolution—ensured robust characterization of both the fine-scale mutations (maternal allele) and the large-scale structural variations (paternal allele). While integrated PacBio and ONT sequencing demonstrates research value for resolving complex RH structural variants, clinical translation faces practical barriers. Current WGS costs (approximately US$2, 000-5, 000 per case) and turnaround times (2–4 weeks) prohibit routine prenatal RH genotyping (28). A tiered approach—initial screening by targeted amplicon sequencing or MLPA, with WGS reserved for unresolved cases—may be more cost-effective (29, 30). For laboratories without third-generation sequencing, long-range PCR across predicted breakpoints with Sanger sequencing remains a viable alternative for structural variant confirmation (31).

Beyond molecular diagnosis, clinical management of D−− pregnancies is challenging due to scarcity of compatible blood (32). Maternal autologous blood offered advantages: immediate availability, immunological compatibility, and repeatability (33). In our case, when no compatible donor was available, we collected autologous blood for the first IUT (pre-collection Hb 10.7 g/dL, Hct 31.8%; 30 mL transfused) (34). A D−− volunteer donor unit was secured through the national rare blood bank for the second IUT. When no additional D−− donors were available for the third and fourth IUTs, we returned to autologous collection (Hb 11.7 g/dL, Hct 34.0% and 34.5%). This sequential approach—prioritizing compatible allogeneic D−− blood when available, with autologous blood as fallback—demonstrates a practical and scalable model for managing rare D−− pregnancies. Maternal hemoglobin (10.7–11.7 g/dL) was maintained with iron, vitamin B12, folic acid, and erythropoietin after each collection (35). The successful outcome at 37 weeks supports feasibility. To our knowledge, this is among the few reported cases of successful management using a combined allogeneic-autologous transfusion strategy for IUT in a D−− pregnancy (16).

This framework is derived from a single case experience and requires prospective validation in larger cohorts. Nevertheless, we propose a preliminary management algorithm emphasizing the central role of the transfusion department (**Figure 6**) (36, 37). Key components include: first-trimester Rh phenotyping and antibody screening; molecular diagnosis, family studies, and rare blood bank registration (36, 37); second-trimester antibody monitoring and weekly MCA-PSV (fetal sampling if ≥1.5 MoM) (21, 38); IUT when fetal Hb <10 g/dL, using D−− donor blood as first-line therapy and maternal autologous blood as an alternative (21, 38); repeated every 2–4 weeks; third-trimester monitoring and delivery planning at 34–37 weeks; and postpartum neonatal monitoring with phototherapy or exchange transfusion as needed (21). This case underscores the central role of the transfusion department in integrating molecular diagnostics, rare blood banking, and transfusion support—a model that may extend beyond D−− to other rare blood group pregnancies. This framework may not apply in resource-limited settings without rare blood registries, in patients with maternal anemia, or in emergencies without time for autologous preparation (37).

**Figure 6:**
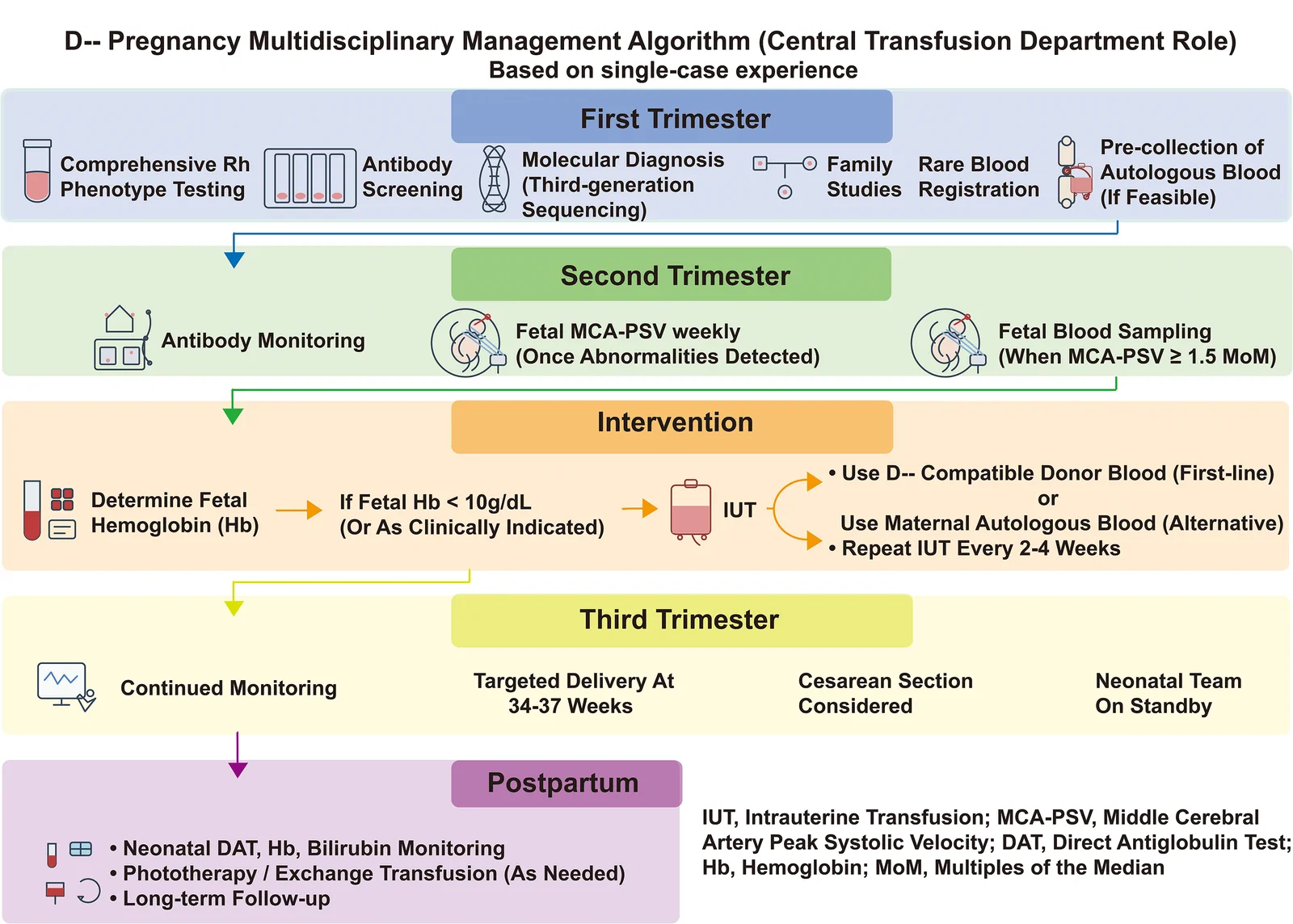
Proposed preliminary management framework based on single-case experience for D−− pregnancies. Flowchart illustrating a suggested multidisciplinary approach from molecular diagnosis through delivery. This framework is proposed as a suggested approach and is not an established clinical algorithm. Clinical decisions should follow institutional guidelines. MCA-PSV thresholds and fetal Hb thresholds are based on published recommendations (SMFM, ACOG, RCOG). In this case, cesarean delivery was performed at 37 weeks due to obstetric indications and to avoid potential birth trauma in a severely anemic fetus. The algorithm is divided into five phases: first trimester (blue), second trimester (green), intervention (orange), third trimester (yellow), and postpartum (purple). IUT, intrauterine transfusion; MCA-PSV, middle cerebral artery peak systolic velocity; DAT, direct antiglobulin test; Hb, hemoglobin; MoM, multiples of the median.

This study has several important limitations. First, it is a single case requiring validation in larger cohorts, and infant long-term follow-up is limited (16). Second, mRNA was unavailable to confirm the effect of c.486 + 1G>A (39). In addition, although fetal hemoglobin was re-measured immediately before the first IUT (8.7 g/dL), the exact trajectory of fetal anemia between diagnostic cordocentesis and the first IUT remains incompletely characterized. Third, the structural rearrangements identified by ONT were not independently validated by Sanger sequencing across the fusion breakpoints (3). Fourth, maternal anti-Rh17 titers were not serially monitored (40), and optical genome mapping was not available to further refine the structural characterization of the paternal allele (3, 10). Finally, the cost and accessibility of whole-genome sequencing may limit routine application, although costs are decreasing (28, 41). Despite these limitations, our findings demonstrate the power of integrated PacBio and ONT WGS for comprehensive RH genotyping and provide a preliminary framework for managing rare D−− pregnancies.

In conclusion, we identified a novel paternal *RHD(1-9)–RHCE(10)–TMEM50A(7-3)–TMEM50A–RHCE(10-8)–RHD(7-2)–RHCE(1)* rearrangement and refined the annotation of the maternal RHCE*02N.03 allele from ISBT C− to C−e− null phenotype. We further demonstrate a sequential allogeneic-autologous transfusion strategy that enabled successful management of severe HDFN with four serial IUTs and delivery at 37 weeks. The proposed management algorithm provides a practical framework for multidisciplinary care of rare D−− pregnancies.

## Data Availability

The datasets presented in this study can be found in online repositories. The names of the repository/repositories and accession number(s) can be found below: https://www.ncbi.nlm.nih.gov/genbank/, PRJNA1521709.
