# Supplementary material for: Compound heterozygous RHCE null genotype underlying D−− phenotype in a Chinese pregnant woman: molecular characterization and sequential transfusion strategy
Source: Front Immunol. 2026 Oct 15;17:2004196. doi: 10.3389/fimmu.2026.2004196 (PMC13716575; doi:10.3389/fimmu.2026.2004196)
Supplement: Supplementary file 1 [file Table1.docx]

**Supplemental Table 1. Serial intrauterine transfusion details and outcome**

| **IUT number** | **Gestational age** | | **EFW, g** | | **Pre-transfusion Hb (g/dL)** | **Pre-transfusion Hct (%)** | **Pre-IUT MoM** | **pRBC Hct (%)** | **Volume (mL)** | **Blood source** | **Post-transfusion Hb (g/dL)** | **Post-transfusion Hct (%)** | **Post-IUT MoM** | **Maternal pre-collection Hb (g/dL)** | **Maternal pre-collection**  **Hct (%)** |
| --- | --- | --- | --- | --- | --- | --- | --- | --- | --- | --- | --- | --- | --- | --- | --- |
| **1** | | **26^+6^ wks** | **864** | **8.7** | | **26.8** | **1.58** | **72.6** | **30** | **Maternal autologous** | **13.4** | **39.3** | **1.01** | **10.7** | **31.8** |
| **2** | | **29^+5^ wks** | **1300** | **7.5** | | **23.1** | **1.45** | **76** | **43** | **D−− volunteer donor** | **13.8** | **41.7** | **0.98** | **—** | **—** |
| **3** | | **33^+1^ wks** | **1928** | **7.7** | | **23.4** | **1.89** | **90.1** | **52** | **Maternal autologous** | **14.5** | **44.2** | **1.06** | **11.7** | **34.0** |
| **4** | | **36 wks** | **2470** | **9.4** | | **29.7** | **1.76** | **82.1** | **53** | **Maternal autologous** | **14.5** | **44.9** | **1.08** | **11.7** | **34.5** |

EFW, estimated fetal weight; Hb, hemoglobin; Hct, hematocrit; IUT, intrauterine transfusion; MoM, multiples of the median; pRBCs, packed red blood cells; wk, week. EFW was determined by ultrasound before each IUT. For the first IUT, pre-transfusion Hb and Hct were measured immediately before the procedure at 26^+6^ weeks (Hb 8.7 g/dL; Hct 26.8%). Diagnostic cord blood sampling on day 3 of hospitalization showed Hb 8.9 g/dL and Hct 27.2%. For maternal autologous transfusions, 200 mL of whole blood was collected per donation and processed into washed irradiated pRBCs; the volume listed in the table represents the final concentrated product transfused to the fetus. After each autologous collection, the patient received oral iron, vitamin B12, folic acid, and subcutaneous erythropoietin (EPO) to support erythropoiesis. Maternal autologous blood (group B) was cross-matched and compatible; D-- volunteer donor blood was also group-compatible. D-- volunteer donor blood was obtained through the national rare blood bank network from an unrelated donor in Shanghai, China.
